# Full-Profile Pharmacokinetic Study of High Dose Baclofen in Subjects With Alcohol Use Disorder

**DOI:** 10.3389/fpsyt.2018.00385

**Published:** 2018-08-23

**Authors:** Nicolas Simon, Romain Moirand, Maurice Dematteis, Régis Bordet, Dominique Deplanque, Benjamin Rolland

**Affiliations:** ^1^Aix Marseille Univ, APHM, INSERM, IRD, SESSTIM, Hop Sainte Marguerite, Service de Pharmacologie Clinique, CAP-TV, Marseille, France; ^2^Univ Rennes, INSERM, INRA, CHU Rennes, Institut NUMECAN (Nutrition Metabolisms and Cancer), CIC 1414, Unité d'Addictologie, Rennes, France; ^3^UFR de Médecine, Université Grenoble Alpes, Grenoble, France; ^4^Service d'Addictologie, CHU Grenoble Alpes, Grenoble, France; ^5^Inserm U1171, Université de Lille, Lille, France; ^6^Inserm CIC1403, CHU Lille, Université de Lille, Lille, France; ^7^Service Universitaire d'Addictologie de Lyon (SUAL), Pôle MOPHA, CH Le VInatier, Bron, France; ^8^Université de Lyon, Inserm U1028, CNRS UMR5292, UCBL, CRNL, Bron, France

**Keywords:** baclofen, alcohol use disorder, pharmacokinetics, human, clinical trial

## Abstract

Baclofen a gamma amino-butyric acid type B (GABA-B) receptor agonist, which has raised some interest for the treatment of alcohol use disorder (AUD), occasionally at dose up to 300 mg/d. We conducted the first full-profile pharmacokinetic study on baclofen in AUD subjects, up to the oral daily dose of 300 mg. Sixty subjects treated for AUD with marketed baclofen were enrolled in a prospective phase-1 study. Participants were divided into four dose groups (1: <60 mg/d; 2: 60–120 mg/d; 3: >120 mg/d-180 mg/d; and 4: >180 mg/d), and they underwent a full-profile pharmacokinetic analysis of baclofen, using a nonlinear mixed effects modeling. The influence of different clinical and biological covariates was assessed in an upward modeling. Fifty-seven participants completed the study (522 observed concentrations collected). Racemic baclofen showed a linear pharmacokinetic profile, corresponding to a one-compartment model, with no influencing clinical or biological factor. The pharmacokinetic parameters of baclofen were (bootstrap 95% confidence intervals): absorption constant (Ka) 1.64 1/h (1.34–2), clearance (Cl/F) 11.6 L/h (10.8–12.3) and volume of distribution (Vd/F) 72.8 L (66.5–80.4) leading to a half-life of 4.4 h. The interindividual variability (IIV) was 44% (19–65), 21% (16–27), and 22% (11–36) for Ka, Cl/F, and Vd/F, respectively. The residual variability was 24% (21–26). No serious adverse event was reported.

**Registration**: EudraCT #2013-003412-46

## Introduction

Baclofen is a gamma amino-butyric acid type B (GABA-B) receptor agonist, which has been used for treating spasticity since the 1970s (1). In this neurological indication, the oral form of baclofen is usually approved for outpatients at the maximum dose of 80 mg/d. In adult, only a few studies have explored the pharmacokinetic profile of oral baclofen in neurological population or healthy volunteers (2–6). Overall, these studies have found a linear elimination of baclofen. For example, Wuis et al. after an oral administration of 40 mg baclofen among healthy volunteers, found a half-life of 6.8 (standard deviation: 0.68) hours (2). The same author team investigated the pharmacokinetics (PK) of baclofen among subjects treated for multiple sclerosis at daily doses between 30 and 80 mg/d, and they confirmed the linear elimination of baclofen at this dose range (3). Moreover, the last study found an important interindividual variability (IIV) of baclofen concentrations among the treated patients.

Since the beginning of the 2000s, an increasing interest has been shown on the therapeutic action of baclofen for alcohol use disorder (AUD). Several randomized clinical trials have investigated the efficacy of a dose of 30 mg/d or 50 mg/d baclofen on different AUD outcomes, with contradictory findings (7–12). Marketed baclofen consists of a racemic mixture, which could impact the efficacy results of these clinical trials. In addition, a few observational studies have suggested that baclofen could have a dose-effect relationship in AUD (13–16). The use of high doses, namely up to 300 mg/day in some patients, has been reported in clinical practice for a few years (13–16). In France, an important prescribing practice of high dose baclofen has been observed since 2008 (17), and has been framed by an official temporary regulatory measure issued by the French drug agency up to the dose of 300 mg/d (18). Two recent randomized clinical trials found no efficacy of baclofen up to the maximum doses of 150 mg/d and 180 mg/d, respectively, on abstinence maintenance (19, 20). However, another trial has found that baclofen, at the maximum dose 270 mg/d, was associated with significantly increased abstinence rates at 12 weeks, compared to placebo (21). Altogether, these findings suggest that, should baclofen be efficacious for drinking reduction and abstinence maintenance, the efficacious dose ranges could occasionally exceed 180 mg/d, which was also suggested by a recent observational study (13). However, the spreading use of high dose baclofen has also been associated with some safety concerns, insofar as baclofen exerts a dose-related sedative action that has may potentiate that of alcohol (21, 22). Overall, this situation warrants exploring the pharmacokinetic features of baclofen in AUD, in particular at high dose ranges, to confirm the linear elimination of baclofen up to the dose of 300 mg/d.

So far, the pharmacokinetics and pharmacodynamics of baclofen have been poorly studied in patients treated for AUD. A recent exploratory study has suggested that baclofen exhibits a linear PK profile in AUD subjects, including at high doses (23). However this study was based on sparse data, and only 3 patients received a dose higher than 120 mg/d. A loss of linearity at high dose could thereby not be excluded, in particular in case of a saturation of elimination or a decrease of absorption. In the first situation, the patient is exposed to a toxicity process, whereas the second consists of a lower than expected exposure. A saturation of renal elimination is theoretically not expected, because glomerular filtration seems to be the dominant mechanism of baclofen elimination (2). However, little is known regarding the absorption process of baclofen, and the use of a possible active transport. Moreover, in a 1992 pilot study that was conducted in 11 neurology patients treated with high dose baclofen, an increased half-life was found, thus suggesting a loss in the linear elimination of baclofen at high doses (4). Studies in healthy volunteers described an absolute bioavailabity of 80% (5, 6), but only with doses of 10 or 20 mg.

Furthermore the article by Marsot and collaborators found an important IIV, which was not explained by individual features such as body weight, gender or biological parameters, i.e., creatinine clearance (23). Secondarily, it has been suggested that two sub-groups of patients could be distinguished, with different speeds of clinical response (24). Such IIV could be explained by differences in drug exposure, meaning that patients receiving a similar dose exhibit different blood and/or concentrations and a variability of the drug efficacy is then expected (23). Otherwise, it can be suggested that the IIV is more likely explained by pharmacodynamic factors. Consequently, a clinical PK study in AUD patients, using appropriate dose ranges, was required to distinguish between these two modalities of variability features.

Xylka® is a new oral formulation of 20 mg baclofen tablets which has been developed with the aim to be labeled for AUD (20). A phase 1 study explored the PK of different dose regimens of this new formulation among subjects who were treated by off-label baclofen for AUD in France. The aims of the study were: (1) to confirm the linear PK of baclofen at high dose ranges; (2) to screen for individual features that may affect baclofen exposure; and (3) to assess the safety of this formulation and the safety of switching from the currently approved forms of oral baclofen into the new one and vice versa.

## Materials and methods

### Study design

This was a Phase I, open-label, steady state study among 60 patients with alcohol use disorder, namely 15 patients in each of the following oral dose ranges: (1) <60 mg/d; (2) 60–120 mg/d; (3) >120–180 mg/d; and (4) >180 mg/d. The total study duration was a maximum of 16 days, including the screening period. Baclofen dispensing and PK sampling were undertaken in a center for clinical investigation (CIC), among three different French university hospitals (Lille, Rennes, and Grenoble).

The different visits were as follows:

- Inclusion Visit: patients were recruited by the investigators within 14 days prior to the PK sampling day (D1).
- Dispensing visit (CIC): 4–7 days prior to D1. Three days before D1, patients had to switch from their marketed baclofen product to the new formulation according to their usual dosing regimen.
- PK sampling visit (D1): patients were admitted to the CIC at least 1 h before the first dose of baclofen and stayed until at least 2 h after the second dose.
- Ending visit (D2): patient were met at the CIC for the last PK sample and collection of patient diaries, and then switched back to the previous marketed baclofen treatment.

### Participants

Sixty subjects were planned to be included in the study. Inclusion criteria were as follows: (1) men and women aged 18 years or more; (2) meeting the DSM-5 criteria for alcohol use disorder; (3) being treated with marketed baclofen for supporting abstinence maintenance or drinking reduction; (4) on a stable dose of baclofen for a least 1 week prior to the drug switch (see below); (5) displaying normal hepatic function or liver cirrhosis with Child Pugh A or B stage; (6) being able to remain abstinent during housing; (7) being affiliated to the French health insurance system; and (8) if females of childbearing potential, being on an efficient birth control method for at least 14 days prior to the first administration of baclofen.

A patient with at least one of the following exclusion criteria was not eligible for enrolment: (1) hypersensitivity to baclofen or to one of the excipients of the new formulation; (2) pregnancy or breast-feeding; (3) liver cirrhosis with Child Pugh C stage, recent hepatic encephalopathy, or current ascites; (4) severely impaired renal function (defined as a creatinine clearance <30 mL/min according to Cockcroft and Gault formula), or severely impaired cardiac or pulmonary function; (5) uncontrolled epilepsy or any history of seizure in non-abstinent patients; (6) infection by Human Immunodeficiency Virus (HIV), Hepatitis B virus (HBV) or Hepatitis C virus (HCV).

### Selection and timing of dose for each patient

Patients took the tested formulation according to their usual dosing regimen regarding, unit and daily oral doses as well as frequency of administration from 3 days before the day of PK sampling. Since baclofen has a t_1/2_ of ~5 h, 3 full days of treatment prior to the start of PK sampling ensured a complete washout from the marketed baclofen product. The number of administrations of baclofen over 24 h depended on the patients' needs. However, the daily schedule must have been fairly established to avoid significant variations from a day to another. Every attempt should be made to include patients having at least 4 h between 2 consecutive intakes in the daytime in order to comply with the PK sampling schedule.

### Drug concentration measurements

Patients were admitted to the Phase I unit (CIC) for PK sampling for a period estimated between 7 and 18 h, depending on the dosing schedule. No food was provided during the hour preceding and following baclofen intakes (N = primary intake and n = secondary intake).

Blood was collected on D1 over 2 consecutive baclofen intakes (N and n). The blood-sampling schedule followed a sparse sampling design given that the results were treated through a population PK analysis. The timing for blood sampling was not absolutely defined, but they were spread over defined time windows for the different patients. The actual blood sampling times were accurately recorded for each patient.

For each patient, the blood sampling occurred during the following time windows:

- Just prior each of the two baclofen doses (preN and pren).
- After the primary administration of baclofen (N): one blood sample during each of the following time windows: 0–1, 1–2, 2–3 h. At least one blood sample was taken between 3 h and the next administration of baclofen (two samples were taken if sufficient time).
- Following the secondary administration of baclofen (n = N+1 or N-1) one blood sample between 0–1 h and another between 1–2 h.

A last blood sample was taken on D2 before one dose (N or n) of baclofen to determine intra- individual variability on residual drug levels.

A total of 9–10 samples were to be taken from each patient.

**Figure 1:**
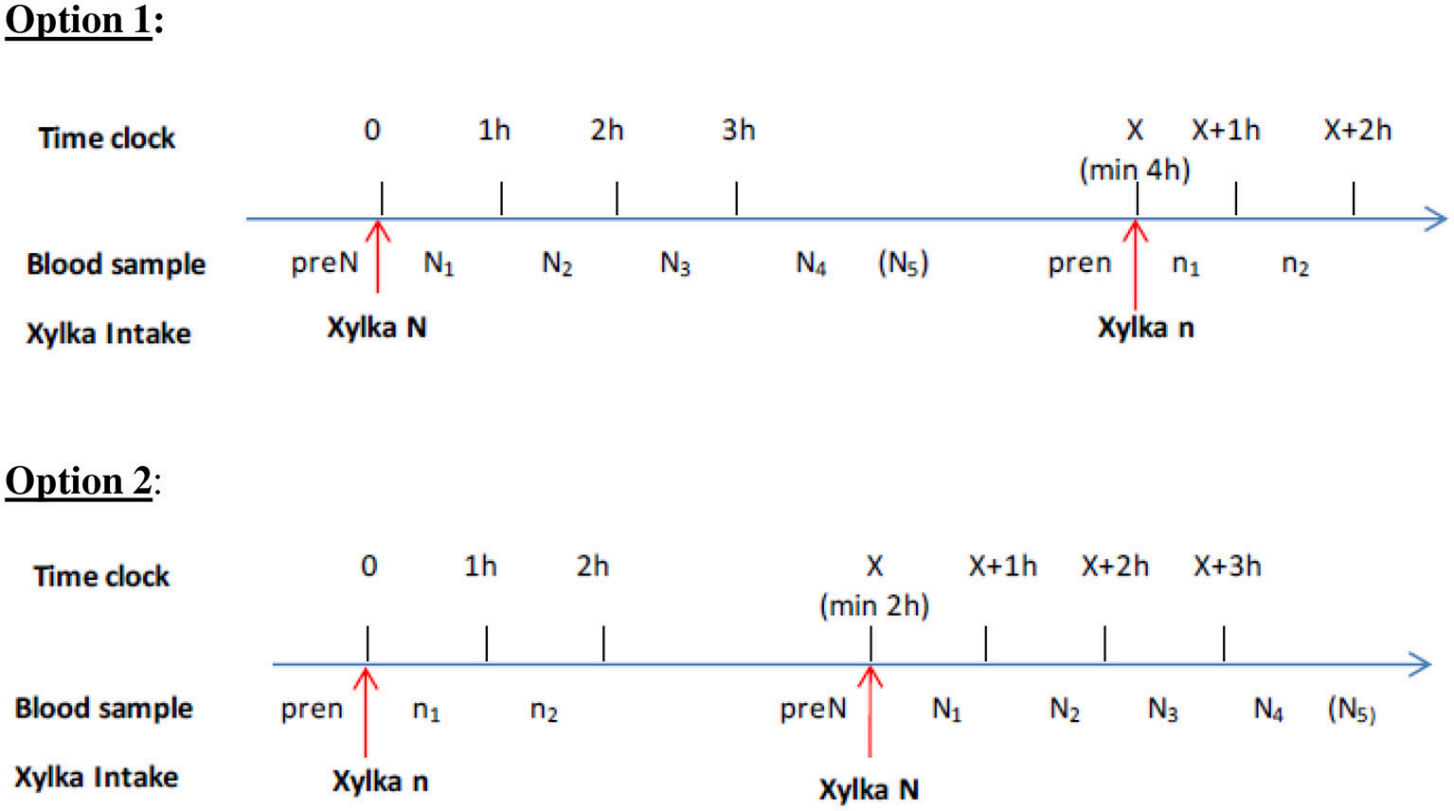
Types of PK sampling strategy, depending on the daily baclofen intake.

Figure 1 shows the two PK sampling schemes used, depending on the daily baclofen intakes.

Baclofen plasma concentrations were determined with a validated method using LC/MS-MS. The method was linear within a calibration range of 5.00–500 ng/mL. Sample reanalysis was conducted on ~10% of the assayed samples to demonstrate reproducibility of the analytical method. The concentration levels from this reanalysis were not used for the pharmacokinetic and statistical evaluations. Standard and quality control samples were distributed through each batch of the study sample assayed. Samples with drug concentrations greater than the upper limit of quantification (ULOQ) of the assay range were diluted with the appropriate drug-free biological fluid and reassayed; those that were below the lower limit of quantification (BLQ) were reported as such by the lab.

### Other data collected

At the inclusion visit, the following data were collected: (1) age; (2) gender; (3) ethnic group; (4) surgical, medical history and concomitant diseases according to the MedDRA; (5) current tobacco smoking (yes/no) and, if yes, number of daily cigarettes; (6) indication of baclofen (abstinence maintenance or controlled drinking); (7) duration of marketed baclofen treatment (in months); and (8) current average weekly use of alcohol.

At the dispensing visit, the total daily dose (mg), daily number of intakes, and mean dose per intake (mg), of baclofen were noted. Concomitant medications were described according to their anatomical therapeutic chemical code (ATC text level 3) and PT (WHO Drug Dictionary, version of March 2013).

All adverse events (AEs) were collected and described according to their system organ class (SOC), as defined in the Medical Dictionary for Regulatory Activities (MedDRA). AEs were defined as “serious” according to the definition of the Food and Drugs Administration (https://www.fda.gov/safety/medwatch/howtoreport/ucm053087.html).

### Population pharmacokinetic analysis

The racemic baclofen mixture was analyzed using a non-linear mixed effects modeling as implemented in NONMEM version 7.3.0 (25). The concepts of this approach known as population pharmacokinetic modeling has been extensively described in the literature (26–28) The First-Order Conditional Estimation (FOCE) with interaction estimation methods was used throughout the modeling. The first step of the base model development was to fit base structural models to the data. Input and output processes was tested as zero, first order or using a Michaelis-Menten equation. Between-subject variability (BSV) of the different pharmacokinetic parameters was estimated with an exponential error model. Several error models (additive, proportional, or both) were investigated to describe residual unexplained variability (RUV). The performance of the models was judged by both statistical and graphic methods (29, 30). Relative standard errors were calculated by use of the COVARIANCE option of NONMEM. The diagnostic plots were the following: observed concentrations (depending variable, DV) vs. population predictions (CPRED) or vs. individual predictions (IPRED) and Normalized predictive distribution error (NPDE) vs. CPRED or vs. TIME. According to these performances, the model that best described the each set of data was defined as “Base Model.”

Once the base model was defined, the influence of different parameters (or covariates), on the pharmacokinetic parameters was explored via an upward model building. These covariates include:

- dose schedule and physiological pieces of information which could impact PK: the daily amount of baclofen (mg) and the inclusion group (GP) but also number of intake by day, sex (1 male, 2 female), body weight (WT in kg), age (yr), body mass index (BMI), lean body mass (LBM).
- and markers of renal and hepatic function: creatinine clearance (CRCL), serum creatinine (CREA), prothrombin time (PT in %), bilirubin, aspartate aminotransferase (AST), and alanine aminotransferase (ALT).

For categorical covariates, if a particular subgroup represents <10% of the overall population, the categories were pooled, as appropriate, to obtain groups with sufficient size for analysis.

The influence of continuous covariates was modeled according to the following equation, using CL for example,

$$\text{CL}={\text{TVCL}}^{*}{\left\{\text{age/median}(\text{age})\right\}}^{\theta \text{age}}$$

where TVCL is the typical value of CL for a patient with the median covariate value and θ age is the estimated influential factor for age (it can be a positive or a negative effect).

The influence of categorical covariates was modeled according to the following equation, using sex effect for example,

$$\text{CL}={\text{TVCL}}^{*}\text{ θ}\_{\text{sex }}^{**}(\text{SEX})$$

where TVCL is the typical value of CL, θ_sex is the estimated influential factor for sex effect and SEX = 0 if male, SEX = 1 otherwise.

When more than 2 categories exit (i.e., the dose “group” covariate has 4 levels), different values of the tested PK parameters was evaluated for each level.

The diagnostic plots described above, the change in objective function, and the change in parameter variability was noted to select those which improved the model prediction. A decrease in the objective function value (OFV) of at least 3.84 (chi-squared distribution with one degree of freedom for *P* < 0.05) relative to the base pharmacokinetic model was required for the addition of a single parameter in the model. Covariates selected during the screening step, was included in a so called “full model.” This was evaluated by a backward elimination procedure in which each covariate was removed in turn from the “full model” and the difference in OFV between the full and each reduced model was examined. An increase in OFV >10.8 (*P* < 0.001) was required to retain the covariate in the final model (the threshold became 16.3 for 3 ddl).

The performance of the model was judged by both statistical and graphic methods. Bootstrap procedures were performed using Wings for NONMEM (www.wfn.sourceforge.net) to evaluate confidence intervals non-parametrically. The final model was used to simulate new data (500 replicates) from the original dataset using the SIMULATION feature in NONMEM (Monte Carlo simulation). These simulated concentrations were then used to construct prediction intervals and were compared with observed data (prediction-corrected Visual Predictive Check).

### Ethics procedure

The study protocol was submitted to and approved by a national ethics committee before the first inclusions (Avis Comité de Protection des Personnes - Amiens 2013/47). In addition, the protocol was declared on an international protocol register prior to the study start (EudraCT Number 2013-003412-46). Written informed consent was obtained from all patients.

## Results

Among the 60 included patients, 3 of them (5%) prematurely discontinued the study and did not receive any dose of baclofen. Thus, the final study sample consisted of 57 patients. The sociodemographic, smoking and drinking patterns, and baclofen intake features of the included subjects are displayed in the Table 1. Only 3 patients were cirrhotic and had a Child-Pugh A score. Two of them were in the dose range >180 mg per day and one in the dose range <60 mg per day.

**Table 1:** Sociodemographics, smoking, and drinking habits, and baclofen dosing features, regarding the subjects included in the study.

**Dose range**						
		**<60 mg/d**	**60–120 mg/d**	**>120–180 mg/d**	**>180 mg/d**	**Total**
		***N* = 10**	***N* = 15**	***N* = 16**	***N* = 16**	***N* = 57**
Age (years)	Mean ± SD	56.75 ± 9.83	44.80 ± 13.21	45.16 ± 11.29	43.97 ± 9.16	46.76 ± 11.73
	Min; Max	42.2; 75.0	23.4; 66.1	25.6; 68.7	33.8; 59.4	23.4; 75.0
Sex	N females (%)	5 (50.0%)	3 (20.0%)	5 (31.3%)	6 (37.5%)	19 (33.3%)
Smokers	N (%)	4 (40.0%)	11 (73.3%)	13 (81.3%)	14 (87.5%)	42 (73.7%)
If smoker, N cig/d	Mean ± SD	22.50 ± 8.66	14.64 ± 5.87	21.92 ± 8.05	16.64 ± 8.39	18.31 ± 8.10
**INDICATION FOR BACLOFEN PRESCRIPTION**						
Abstinence maintenance	N (%)	8 (80.0%)	8 (53.3%)	8 (50.0%)	9 (56.3%)	33 (57.9%)
Drinking reduction	N (%)	2 (20.0%)	7 (46.7%)	8 (50.0%)	7 (43.8%)	24 (42.1%)
Duration of marketed baclofen treatment (months)	Mean ± SD	11.03 ± 10.31	11.54 ± 11.31	10.80 ± 5.73	16.10 ± 11.97	12.72 ± 10.19
	Min; Max	0.2; 26.4	0.3; 35.4	4.2; 20.8	1.7; 39.6	0.2; 39.6
Current alcohol abstinence	N (%)	6 (60.0%)	10 (66.7%)	7 (43.8%)	5 (31.3%)	28 (49.1%)
If no, average weekly alcohol consumption (g alcohol/week)	Mean ± SD	130.8 ± 235.4	82.0 ± 54.0	137.8 ± 175.8	216.8 ± 255.9	158.1 ± 202.9
	Min; Max	0 ; 49.0	0 ; 15.0	0 ; 60.0	0 ; 84	0 ; 84
Baclofen total daily dose (mg)	Mean ± SD	37.00 ± 9.49	93.33 ± 21.93	163.13 ± 17.78	250.63 ± 39.41	147.19 ± 81.91
	Min; Max	30.0; 50.0	60.0; 120.0	130.0; 180.0	190.0; 300.0	30.0; 300.0
Baclofen mean number of daily intakes	Mean ± SD	2.80 ± 0.63	3.40 ± 0.74	3.44 ± 1.15	3.94 ± 1.48	3.46 ± 1.13
	Min; Max	2.0; 4.0	2.0; 5.0	2.0; 7.0	2.0; 8.0	2.0; 8.0
Baclofen mean dose per intake (mg)	Mean ± SD	13.92 ± 5.24	28.49 ± 9.11	50.47 ± 11.48	70.16 ± 23.19	43.80 ± 25.26
	Min; Max	10.0; 25.0	20.0; 50.0	25.7; 75.0	37.5; 125.0	10.0; 125.0

N, number;

SD, standard deviation;

*cig, cigarette*.

No outlier was visually identified, and the modeling was thus performed on all available concentrations (i.e., 522 observations, 57 patients).

The dataset was best described using a one-compartment model, with first order absorption and elimination (ADVAN2, TRANS2 subroutine). The PK model was parameterized in term of clearance (CL/F), volume of distribution (Vd/F), and absorption rate constant (Ka). Between subject variability (BSV) of the different PK parameters and residual unexplained variability (RUV) were estimated with an exponential model. The estimates of the shrinkage for CL/F, Vd/F, and Ka were 0.05, 0.22, and 0.37, respectively for baclofen, suggesting that individual estimates for CL/F and Vd/F were robust but less reliable for Ka. None of the covariates tested were able to improve the fit, to decrease the intra-individual variability (IIV) or to decrease significantly the objective function of the model. Thus, the model without any covariate (i.e., basic model) was considered as the final model.

The performance of the model was judged satisfactory as depicted by the diagnostic plots (see Figure 3). The plots describing population (CPRED) or individual (IPRED) predicted concentrations vs. observed concentrations showed a good correlation. The normalized predictive distribution error (NPDE) vs. time or CPRED did not show any trend of a bias (Figure 3). The values were mostly between −4 and +4 and were evenly distributed around “0.” The estimation of the PK parameters is shown in Table 2.

**Table 2:** Population pharmacokinetic parameters of baclofen.

**Parameters**	**Estimation**	**Rse**	**Bootstrap**
		**(%)**	**95% CI**
CL/F (L/h)	11.6	3	10.8–12.3
Vd/F (L)	72.8	5	66.5–80.4
Ka (1/h)	1.64	10	1.34–2.00
**BETWEEN SUBJECT VARIABILITY**			
ω(CL/F)	0.21	14	0.16–0.27
ω(Vd/F)	0.22	31	0.11–0.36
ω(Ka)	0.44	24	0.19–0.65
**RESIDUAL VARIABILITY**			
σ exponential	0.24	5	0.21–0.26

*Rse, relative standard error; CL/F, clearance; Vd/F, volume of distribution; Ka, absorption constant; CI, confidence interval*.

**Figure 2:**
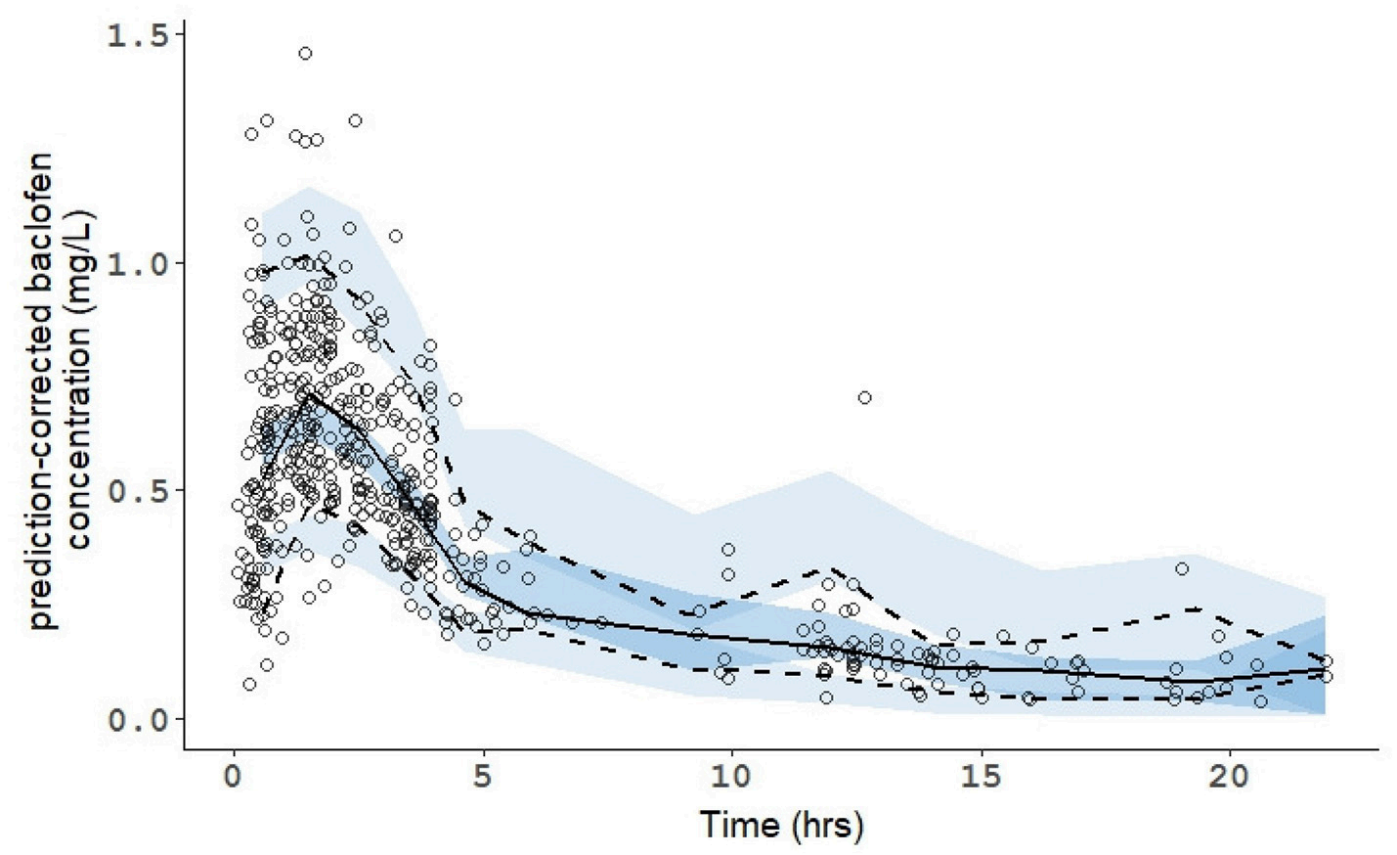
Prediction-Corrected Visual Predictive Check (pcVPC) of the population pharmacokinetic model for the racemic mixture of baclofen. The line and the dashed lines are the observed percentiles (50, 5, and 95th percentiles). The shaded fields represent a simulation-based 95% confidence interval for the 5, 50, and 95th percentiles. The observed plasma concentration (prediction corrected) are presented by circles.

The robustness of the estimation was confirmed by the bootstrap method. All estimated parameters were within the 95% confidence interval which demonstrated the stability of the final models. Consistently, the prediction-corrected Visual Predictive Checks confirmed that simulated data are consistent with observed data (Figure 2).

Safety features were also assessed during the study. 21 patients (36.8%) presented at least one AE; 37 AEs occurred in total. The System Organ Class (SOC) details of the observed AEs are reported in Table 3. No serious AE was observed during the study. No significant changes were observed for vital signs between the dispensing visit and the assessment visit or between the assessment visit and the end of study visit when relevant. No significant values or differences between dose ranges were observed in the clinical laboratory data.

**Table 3:** Most frequent adverse events displayed according to System Organ Class and Preferred Term.

**Dose range**						
		**<60 mg/d**	**60–120 mg/d**	**>120–180 mg/d**	**>180 mg/d**	**Total**
**System organ class**	**Preferred term**	***N* = 10**	***N* = 15**	***N* = 16**	***N* = 16**	***N* = 57**
**At least one AE**		4 (40.0%)	4 (26.7%)	7 (43.8%)	6 (37.5%)	21 (36.8%)
**Nervous system disorders**		2 (20.0%)	1 (6.7%)	1 (6.3%)	4 (25.0%)	8 (14.0%)
	Headache	1 (10.0%)	–	1 (6.3%)	2 (12.5%)	4 (7.0%)
	Dizziness	1 (10.0%)	–	–	1 (6.3%)	2 (3.5%)
	Paresthesia	–	1 (6.7%)	–	1 (6.3%)	2 (3.5%)
**Gastrointestinal disorders**		–	1 (6.7%)	1 (6.3%)	3 (18.8%)	5 (8.8%)
	Nausea	–	–	–	3 (18.8%)	3 (5.3%)
	Vomiting	–	1 (6.7%)	1 (6.3%)	–	2 (3.5%)
**Psychiatric disorders**		1 (10.0%)	1 (6.7%)	2 (12.5%)	–	4 (7.0%)
	Anxiety	1 (10.0%)	1 (6.7%)			2 (3.5%)
	Sleep disorder			2 (12.5%)		2 (3.5%)

## Discussion

The objectives of the study were: (1) to confirm the linear PK of baclofen with a new formulation; (2) to screen for individual features that may affect baclofen exposure; and (3) to assess the safety of a new oral formulation and the safety of switching from currently approved forms of oral baclofen into the new oral formulation and vice versa.

The concentrations were appropriately described by a linear one-compartment model with first order input, clearance, and a volume of distribution. More complex models did not improve the fit. The final model displayed no formal bias as shown in both the diagnostic plots (see Figure 1) and the prediction-corrected Visual Predictive Checks (see Figure 2). There was no saturation effect in the absorption, distribution, metabolism, or elimination processes. Consequently, the final model demonstrates the linear kinetics of baclofen over the tested range. This main finding is consistent with what was suggested in previous studies (23, 24). However, before the present study, this was never investigated using richer data, in a full-profile pharmacokinetic study.

**Figure 3:**
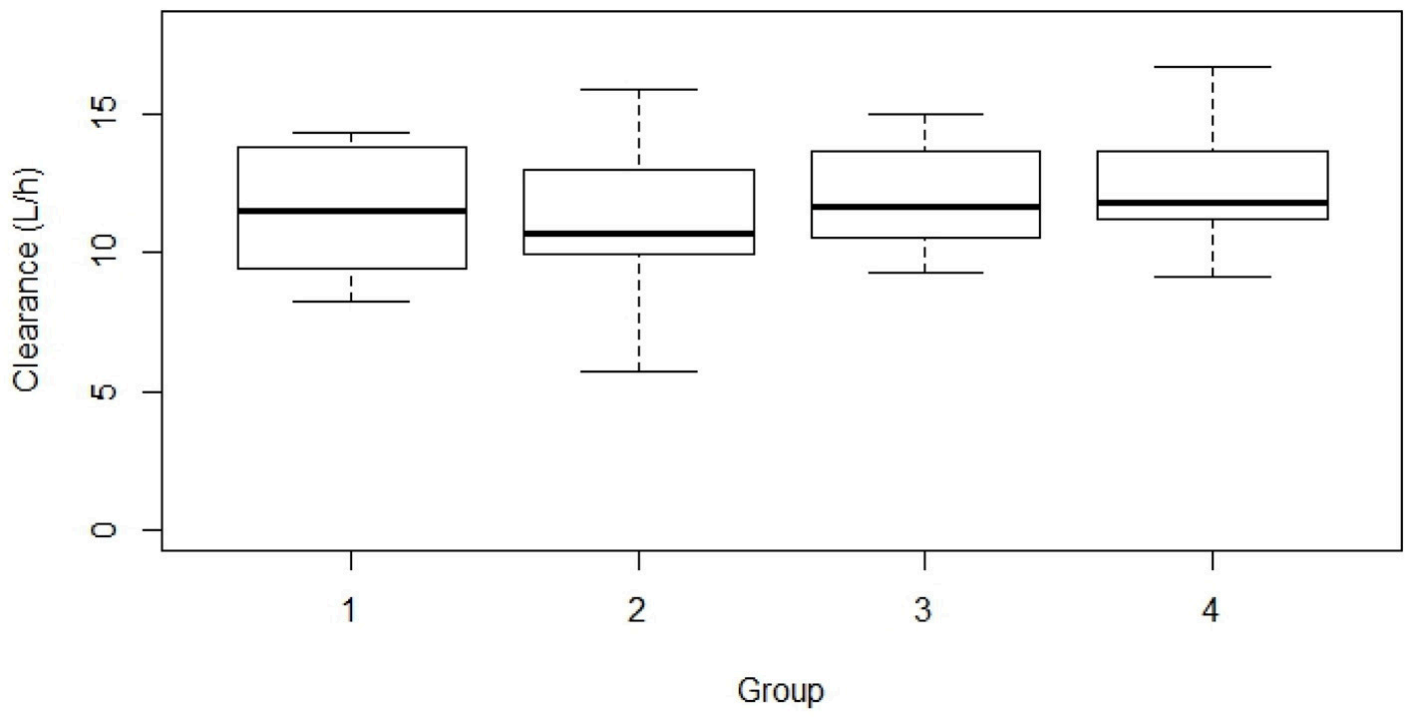
Clearance of baclofen according to the group. (1) <60 mgpd; (2) 60–120 mgpd; (3) >120–180 mgpd; and (4) >180 mgpd.

As part of the second objective, we explored the covariates that could influence the PK characteristics of baclofen. However, none of the covariate tested were able to explain the IIV or improve the fit of the initial model. Consequently, the final model did not include any covariate. In particular, the clearance was not influenced by the dose used (see Figure 3). This result means that no change in metabolism/elimination was observed for baclofen up to 300 mg/day. Furthermore, the IIV were 21, 22, and 44% for CL/F, Vd/F, and Ka, respectively. These values are in accordance with previous results which did not find a wide inter-individual variability of the exposure (23). The blood concentrations of baclofen exhibited a linear PK corresponding to a one-compartment model without any influencing covariate. For the doses used in this study (30–300 mg/day), no accumulation after once daily dosing was noted, and no adjustment was required whatever be the gender, body weight, or creatinine clearance.

This set of findings has some clinical and therapeutic implications. The efficacy of high dose baclofen is still under debate, based on the most recent published clinical trials (19, 20, 31). However, the results of some observational studies have suggested that the effectiveness of baclofen was very variable among individuals (13–17), which could be related to a variability of blood exposure. Our study confirms the IIV of baclofen PK parameters (21% for CL/F and 22% for Vd/F), but this variability cannot fully explain the variability of baclofen effectiveness. Other mechanisms should thus be explored to address this variability, including the mechanisms involved in the crossing of the blood-brain barrier, or pharmacodynamics features that may affect the action of baclofen on the GABA-B receptors. This statement is also applicable to some baclofen-induced AEs, for example sedation, whose occurrence is dose-related but also highly variable according to individuals (23). In the light of the present results, such clinical variability cannot be explained by PK features.

The third and last objective of the study was to assess the tolerability of a new oral formulation, as well as the safety of switching from currently approved forms of oral baclofen into a new one and reciprocally. Overall, baclofen was well tolerated, with no serious AEs occurring during the study. The types of non-serious AEs reported with the new formulation were similar those reported with the off-label use of marketed forms of baclofen for alcohol dependence (22). The switching stages from marketed forms of baclofen to the tested formulation, and reciprocally, were associated with few and mild TEAEs. However, the collecting of safety data in this protocol only consisted of assessing whether safety issues occurred during the study. We did not intend to paint an exhaustive picture of baclofen safety, which has been addressed elsewhere (32). Moreover, other PK studies on baclofen have reported that safety issues could occur in case of impaired renal function (33), and no such patient was recruited in our study. In addition, concerns about suicidal ideations have been raised with the use of high dose (34–36), even if no clear baclofen causality has been found so far (37). However, no suicidal ideation was reported during our study.

In total, this study confirmed that the racemic mixture of baclofen showed a linear PK profile, corresponding to a one-compartment model with no significant influencing covariate. Overall, baclofen was well tolerated, and switching from marketed forms of oral baclofen into the new one, or the opposite, did not induce substantial safety issues.

## Author contributions

NS, BR, and DD, wrote the study design. BR, RM, and MD, recruited the subjects. NS performed the statistical analyses. NS and BR wrote the first draft of the manuscript. NS, BR, DD, RB, RM, and MD contributed to and have approved the final version of the manuscript.

### Conflict of interest statement

Ethypharm was the entire funder of the study. Ethypharm was involved in writing the study protocol, in collaboration with NS, BR, and DD. Ethypharm also took part in the data analyses, in collaboration with NS. Ethypharm was not involved in the writing of the manuscript, but gave their permission for submission. The remaining author declares that the research was conducted in the absence of any commercial or financial relationships that could be construed as a potential conflict of interest.
